# A novel multiplex qPCR targeting 23S rDNA for diagnosis of swine dysentery and porcine intestinal spirochaetosis

**DOI:** 10.1186/s12917-016-0939-6

**Published:** 2017-02-07

**Authors:** Anna Borgström, Simone Scherrer, Constanze Kirchgässner, Sarah Schmitt, Daniel Frei, Max M. Wittenbrink

**Affiliations:** Institute of Veterinary Bacteriology, Vetsuisse Faculty, University of Zurich, Winterthurerstrasse 270, CH 8057 Zurich, Switzerland

**Keywords:** Swine dysentery, *Brachyspira hyodysenteriae*, 23S rDNA, Multiplex Real-time PCR, Swabs

## Abstract

**Background:**

A multiplex qPCR targeting a 128 bp region on the 23S rDNA gene was developed for detection of *Brachyspira (B.) hyodysenteriae* and *B. pilosicoli*, the agents of swine dysentery (SD) and porcine intestinal spirochaetosis (PIS), together with a triplet of apathogenic *Brachyspira* spp. (*B. innocens, B. intermedia, B. murdochii*) in porcine feces. The multiplex qPCR was evaluated against a duplex PCR (La et al., J Clin Microbiol 41:3372–5, 2003).

**Results:**

Using DNA extracted from fecal culture, the multiplex qPCR showed excellent agreement with the duplex PCR (κ = 0.943 and 0.933). In addition, thanks to the three probes whereof one detecting the apathogenic *Brachyspria* spp., a more diversified overview of the brachyspiral flora in porcine fecal samples can be delivered as a part of the routine diagnostic. The multiplex qPCR with a limit of detection of 5–10 genomic equivalents (GE) per reaction (6 × 10^2^ GE per gram) allows reliable detection of *Brachyspira* species directly from fecal swab DNA. In line with this, analysis of 202 fecal swabs in comparison with culture-based qPCR showed a high agreement for the causative agents of SD (*B.hyodysenteriae:* κ = 0.853, sensitivity 87% specificity 98%).

**Conclusion:**

The novel multiplex qPCR is robust and has a high analytical sensitivity and is therefore suitable for high-throughput screening of porcine fecal swabs for the causative agents of SD. This assay can therefore be used for the direct proof of the pathogenic *B*. spp. in fecal swabs within the scope of a monitoring program.

**Electronic supplementary material:**

The online version of this article (doi:10.1186/s12917-016-0939-6) contains supplementary material, which is available to authorized users.

## Background

*Brachyspira* (*B.) hyodysenteriae* is the causative agent of swine dysentery (SD), a severe mucohaemorrhagic diarrheal disease of weanling to finishing pigs [1]. SD occurs worldwide and causes significant economic loss in affected pig production systems. Aside from *B. hyodysenteriae*, other *Brachyspira* species are found in the porcine intestine. *B. pilosicoli* causes the porcine intestinal spirochaetosis (PIS), an enteric disease in weaning pigs clinically resembling SD, but with milder symptoms [2]. To date, three additional species have been described in pigs, namely *B. innocens, B. intermedia* and *B. murdochii*. Although *B. intermedia* has been suspected to cause colitis and diarrhea in swine [3–5], experimental infections of pigs failed at all points [6, 7]. Therefore, *B. innocens, B. intermedia* and *B. murdochii* are still considered as only mildly pathogenic or as commensals in pigs [8, 9]. PIS has been observed in Switzerland for over a decade [10]. However, etiologically confirmed cases of SD were diagnosed for the first time as late as 2008 [11]. Since then the agent is spreading throughout Switzerland. Currently *B. hyodysenteriae* is diagnosed in nearly 2% of all pig herds served by the Swiss Pig Health [12]. To further monitor the spreading and clinical significance of *B. hyodysenteriae* in the Swiss pig population, large scale analyses on porcine fecal specimens for *Brachyspira* are necessary.

Standard procedure for the bacteriological diagnosis of SD/PIS consists of selective culture of anaerobic spirochetes from clinical specimens (feces, colonic tissue) [13] and subsequent classification of spirochetal isolates to the species level by PCR targeting the brachyspiral 16S rDNA and NADH Oxidase (*nox*) gene [14], respectively. However, culture-coupled PCR to detect pathogenic *Brachyspira* often requires six days or longer to be completed [15], and its workflow can be tedious compared to modern real-time PCR assays, a major drawback of this approach when a large series of specimens should be analyzed. In two previous studies comparing direct fecal-PCR with culture coupled PCR it was found that direct fecal PCR assays are nearly a 100-fold less sensitive than culture coupled PCR, and are, therefore, not suitable for the reliable identification of infected pigs [15, 16]. Moreover, conventional PCR assays do not contain internal controls making false negative result a possible problem, especially when analyzing difficult fecal samples. Thus, it is of particular interest to improve the analytic sensitivity of PCR detection methods for large scale analyses of porcine fecal specimens for *Brachyspira.* In the present study, we describe the evaluation of a novel 23S rDNA multiplex qPCR on a diversified panel of fecal specimens from pig herds suspected of SD.

## Methods

### *Brachyspira* strains and growth conditions

Twelve reference strains representing six *Brachyspira* species were included in this study: *B. hyodysenteriae* B78 ATCC 27164, *B. hyodysenteriae* B204 ATCC 31212, *B. hyodysenteriae* WA-1 ATCC 49526, *B. pilosicoli* P43/6/78 ATCC 51139, *B. pilosicoli* 102/06^1^ *B. innocens* B256 ATCC 29796, *B. innocens* 8244/05^2^, *B. murdochii* 56-150 ATCC 51284, *B. murdochii* 403-2x/06^2^, *B. intermedia* PWS/A ATCC 51140, *B. intermedia* 863/06^2^ and *B. alvinipulli* 91-1207/C1 ATCC 51933. The strains were cultured on selective Tryptose Soy Agar (TSA) as described elsewhere [17, 18].

### Fecal samples

**Fig. 1:**
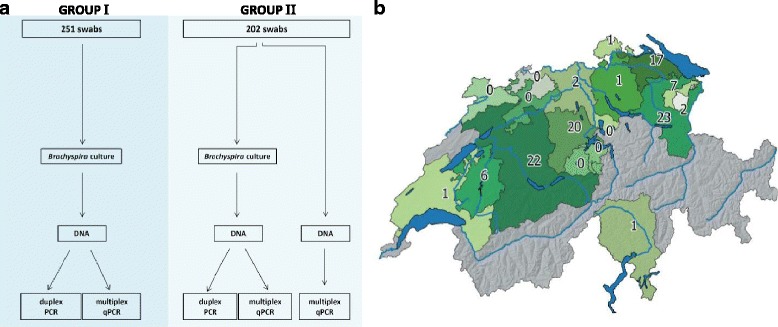
Sample groups and origin of collected samples. The 453 samples in this study were divided into two different samples groups (1–2). Group 1 consisted of cultured samples compared with the duplex PCR, Group 2 contained DNA samples directly extracted from the swabs without the culture step, and the same samples after culture of the swabs. All samples results were compared between the multiplex qPCR and the conventional duplex PCR (**a**). Map of the Swiss cantons from which the samples were collected. Colored cantons were included in the study and the total of *B.hyodysenteriae* positive samples detected with the multiplex qPCR are marked in each region (**b**). For 11 of the *B. hyodysenteriae* positive samples data about origin was not provided

Rectal swabs were collected from feeder pigs on 178 farms from 18 different Swiss Cantons (Fig. 1). All samples were routine submissions during a SD monitoring program and were collected between August 2009 and September 2015. Swabs with fecal matter were inserted into Amies transport medium in airtight screw cap plastic vials (VWR, Dietikon, Switzerland) and transported to the laboratory under cooled conditions within 18 h. Swabs were cultured for *Brachyspira* spp. as cited above. Afterwards, the swabs were stored at −20° until DNA extraction. Areas of weak or strong beta-hemolysis on the TSA plates were examined for spirochetal growth by dark-field microscopy of surface scrapings resuspended in a small volume of 0.15 M NaCl. Spirochetal isolates were subcultured anaerobically on Colombia sheep blood agar (Oxoid, Pratteln, Switzerland). For PCR analyses, spirochetal surface growth was directly transferred from Colombia sheep blood agar into 1 ml sterile distilled water.

### Sample groups

Overall, a panel of 453 swabs with a positive culture of *Brachyspira* spp. was arbitrarily selected from diagnostic samples. Samples were divided into two groups for comparison of culture and PCR methods as outlined in Fig. 1. Group 1 consisted of 251 fecal swabs that were analyzed by culture coupled duplex PCR and multiplex qPCR. Group 2 contained 202 swabs for the comparison of culture coupled PCR assay and direct fecal multiplex qPCR.

### DNA extraction

For DNA extraction the total bacteria lawn from the agar plate was harvested in 1 ml sterile distilled water and with the use of a drigalski spatula. DNA was extracted from *Brachyspira* reference strains as well as fecal isolates by using the InstaGene Matrix (Bio Rad, Cressier, Switzerland) according to the manufacturer’s instructions. For DNA extraction of fecal swabs, first an overnight incubation step at 56 °C in ATL buffer supplemented with proteinase K (60 mAU/ml, Qiagen, Basel, Switzerland) was carried out, followed by DNA extraction with the QIAcube system and the Cador Pathogen DNA extraction protocol (Qiagen) with additional Carrier RNA (1 μg per 100 μl, Qiagen) added to the VXL buffer. The DNA was measured at 260 nm using a NanoDrop 2000c Spectrophotometer (Thermo Scientific, Reinach, Switzerland) for the determination of concentration, diluted to 20 ng/μl and stored at −20 °C until use.

### Duplex PCR

The duplex PCR for the detection of *B. hyodysenteriae* and *B. pilosicoli* was performed as described by La et al. [14], (Table 1) on a Veriti® 96 -well thermal cycler (Applied Biosystems, Life Technologies, Zug, Switzerland). Primers were synthesized by Microsynth (Balgach, Switzerland). PCR products were analyzed by QIAxcel capillary electrophoresis with the screening cartridges (Qiagen).

**Table 1:** Oligonucleotide primer and probe sequences with their respective reporter dye and quencher used in this study

Primer or probe name	Target	Concentration	Sequence 5′→3′	Amplicon
Multiplex qPCR				
Primer for	23S rDNA	0.4 μM	TTCGATGGAATGACACAGATTGT	128 bp
Primer rev	23S rDNA	0.4 μM	CCGAAAGCCCAGTCACTATC	
Probe_hyo	*B.hyodysenteriae*	100 nM	6-FAM-CCTTAACCTTAAAGAAGCAAGCAT(BHQ-1)TTGACTCACCTCAAG-SpacerC3	
Probe _pilo	*B.pilosicoli*	100 nM	Yakima Yellow-AGGTGATGGTTATCCTCGTCGAAT-BHQ-1	
Probe_ apathogen	*B. intermedia/* *B. innocens/* *B. murdochii*	25 nM	Dragonfly Orange-CCTCAACCTTAAAGCAACAAGCAT(BHQ-2)TTTACTCATCACAAG-SpacerC3	
eGFP for	enhanced GFP	0.2 μM	GACCACTACCAGCAGAACAC	177 bp
eGFP rev	enhanced GFP	0.2 μM	GAACTCCAGCAGGACCATG	
Probe_eGFP	enhanced GFP	25 nM	ATTO 647N-AGCACCCAGTCCGCCCTGAGCA-BHQ-3	
Duplex PCR [12]				
*B.hyo*_for	*nox*	0.5 μM	ACTAAAGATCCTGATGTATTTG	345 bp
*B.hyo*_rev	*nox*	0.5 μM	CTAATAAACGTCTGCTGC	
*B.pilo*_for	16S rDNA	0.17 μM	AGAGGAAAGTTTTTTCGCTTC	823 bp
*B.pilo*_rev	16S rDNA	0.17 μM	GCACCTATGTTAAACGTCCTTG	

### Development of the multiplex real-time PCR

#### Primers, probes and PCR settings

Primers and probes were selected using CLC Main Workbench software (Vers. 7.5.1, Qiagen) from alignments of the available sequences of the 23S rDNA gene (NCBI databank). The selected primers amplify the same 128-bp fragment on the 23S rDNA gene of all five different *Brachyspira* spp. (*B. hyodysenteriae*, *B. pilosicoli*, *B. intermedia*, *B. innocens*, *B. murdochii*). On the 128-bp amplicon, target sequences for three DNA probes specific for (i) *B. hyodysenteriae*, (ii) *B. pilosicoli* and (iii) a triplet of three apathogenic species (*B. intermedia*, *B. innocens*, *B. murdochii*) were identified (Table 1, Additional file 1: Figure S1). BLAST searches of both primer and probe sequences were conducted to confirm gene and species specificity. Oligonucleotide primers were synthesized by Microsynth (Balgach, Switzerland). DNA probes were provided by Eurogentec S.A. (Seraing, Belgium). All probes are quenched by black-hole non-fluorescent quenchers either at the 3′-end (probe_pilo) or coupled internally (probe_hyo, probe_apathogen, Table 1). Rox dye, as a component of the Fast Universal PCR Master mix (Applied Biosystems) was used as passive reference for normalization purposes.

An internal amplification control (IAC) was added to each reaction well for signalling presence of PCR inhibitory substances. Five femtogram (fg) of a 712 base pair fragment of the enhanced green fluorescent protein (eGFP) gene was used, and a 177 base pair long amplicon [19] was generated by amplification with specific eGFP forward and eGFP reverse primers and detected by the eGFP-probe with ATTO 647 N reporter dye (Table 1). DNA samples from three ATCC reference strains (*B. hyodysenteriae* 4000 fg, *B. pilosicoli* 40 fg, *B. intermedia* 4000 fg) were used as positive controls in each PCR run. The PCR was performed on an ABI7500 Instrument (Applied Biosystems) using the TaqMan® Fast Universal PCR Master Mix (2x), no AmpErase® UNG (Applied Biosystems). Each reaction contained 5 μl master mix, 1 μl primer mix with eGFP primer (2 μM) and 23S rDNA primer (4 μM), 1 μl of probe mix consisting of 100 nM Yakima Yellow labeled probe, 100 nM 6-FAM labeled probe, 25nM Dragonfly Orange labeled probe, 25 nM ATTO 647 N labeled probe, 1 μL internal control eGFP DNA (5 fg), 2 μl template DNA (20 ng/μl). The cycling conditions were 20 s at 95 °C, followed by a two-step cycling stage of 45 cycles of 15 s at 95 °C and 60 s at 62 °C. The ramp in the cycling stage was set at 80% in order to ensure an efficient hybridization of primers and probes to the template DNA. Samples were considered positive when presenting a typical amplification curve with a C_t_ value of ≤38. The concentration of the IAC was adjusted to have a C_t_ value around 27 to allow reliable amplification of the target gene. Analyses of samples with IAC C_t_ values >32 were repeated after reduction of PCR-inhibitory substances by 1:2 and 1:10 dilution.

#### Analytical sensitivity and specificity

Analytical sensitivities of the multiplex qPCR were determined by plotting standard curves (C_t_ values against quantified genomic equivalents, GE) in ten-fold dilution from 4 × 10^7^ to 4 × 10^3^ GE. With an estimated genome size of 3 Mb for *B. hyodysenteriae,* 2.6 Mb for *B. pilosicoli*, and 3.2 Mb for the apathogenic species [20], the following DNA quantities for 1 GE were calculated: *B. hyodysenteriae* 3.3 fg, *B. pilosicoli* 2.8 fg, and *B. intermedia/B. murdochii/B. innocens* 3.5 fg. The slope of the linear relationship of this curve was used to calculate the amplification efficiency [21]. The minimum detectable bacterial concentrations were determined as the quantification limits of the multiplex qPCR. For each reference strain twelve replicates of samples containing 50, 25, 15, 10, 5, 2, 1 GE per 10 μl PCR reaction were analyzed. Primers and probes were confirmed specific for each *Brachyspira* species in the monoplex real-time set up. The multiplex format was optimized in terms of probe and primer concentrations and annealing temperature by comparing the efficiencies of PCR runs. To determine the specificity of the optimized multiplex qPCR, a variety of DNA mixtures from different *Brachyspira* spp. as well as a variety of 26 spirochetal and non-spirochetal bacteria were used (Additional file 2: Figure S2).

### Data analysis/statistics

The agreement between the conventional duplex PCR and the multiplex qPCR of swabs or cultured isolates was evaluated by Cohen’s kappa index using the IBM SPSS Statistics 22 Software. The strength of agreement was ranked accordingly: Poor (<0.00), Slight (0.00–0.20), Fair (0.20–0.40), Moderate (0,41–0.60), Substantial (0.61–0.80), Almost perfect (0.81–1.00) [22]*.*

## Results

### Sensitivity and specificity of the multiplex qPCR

The efficiency of the multiplex qPCR was determined by the use of serial dilution standard curves. The linear correlation coefficient r^2^ for the three different targets within 23S rDNA was between 0.98 and 0.99, showing a significant linearity of the multiplex qPCR assay (Additional file 3: Figure S3). The amplification efficiencies were 97% for *B. pilosicoli*, 92% for *B. hyodysenteriae* and 103% for *B. intermedia.* Furthermore, the limit of detection (LOD) of each target was evaluated for the multiplex qPCR. The LOD was defined as the lowest number of GE which gave rise to a detectable signal in all of the 12 replicate samples. The LODs were set at 10 GE for *B. hyodysenteriae* and *B. pilosicoli* (equal to 33 and 28 fg DNA), while the detection limit for the triplet of apathogenic *Brachyspira* spp. was set at 5 GE (17.5 fg DNA). The sensitivity and specificity of the assay was calculated for the two pathogens in both sample groups (Table 2).

**Table 2:** Sensitivity and specificity calculated for the multiplex qPCR assay, compared to the gold standard duplex PCR

Method		Total samples (n)	Target spp.	Sensitivity (%) (95% CI)	Specificity (%) (95% CI)
Group 1	multiplex qPCR	251	*B.hyodysenteriae*	98 (86–97)	97 (94–99)
			*B.pilosicoli*	93 (93–100)	96 (96–100)
Group 2	multiplex qPCR	202	*B.hyodysenteriae*	100 (90–100)	98 (94–100)
			*B.pilosicoli*	94 (87–98)	98 (94–100)
	swabs^a^	202	*B.hyodysenteriae*	87 (72–96)	98 (94–99)
			*B.pilosicoli*	65 (54–76)	96 (91–99)

^a^Swab-coupled multiplex qPCR analysis compared with culture-coupled multiplex qPCR

The performance of the optimized multiplex qPCR with different strains of the targeted *Brachyspira* taxons demonstrated no unspecific reactions with other bacteria spp. or cross amplification between the different target species. The multiplex qPCR was shown to simultaneously detect all three *Brachyspira* targets in one reaction and the C_t_ values did not show any significant shift as a result of the multiplex setup compared with those of the monoplex reactions (data not shown). No-template controls as well as the panel of 26 heterologous bacteria consistently scored negative.

### Evaluation of the multiplex qPCR

#### Comparison of the duplex and multiplex PCR using DNA from culture samples

DNA from a total of 251 cultured harvests was analyzed with the multiplex qPCR in parallel with the conventional duplex PCR (Fig. 1, Group I). The multiplex qPCR identified a total of 76 out of 251 samples (30.3%) *B.hyodysenteriae*-positive. One sample was found negative for *B.hyodysenteriae* with the multiplex qPCR while classified as positive with the duplex PCR. In contrast, the multiplex qPCR found five new *B.hyodysenteriae-*positive samples which were not detected by the duplex PCR. The multiplex qPCR detected 93 of 251 samples (37.1%) as *B.pilosicoli* positive, while seven samples were classified as negative in the multiplex qPCR although being positive in the duplex PCR. Regarding the detection of *B.hyodysenteriae* and *B.pilosicoli* both PCR methods showed a significant agreement with κ-values of 0.943 and 0.933 respectively (Table 3).

**Table 3:** Result from the multiplex qPCR analysis of culture derived DNA in Group 1 (A). Agreement between the multiplex qPCR and the duplex PCR calculated with the kappa test (B). The strength of agreement was ranked accordingly: Poor (<0.00), Slight (0.00–0.20), Fair (0.21–0.40), Moderate (0,41–0.60), Substantial (0.61–0.80), Almost Perfect (0.81–1.00). B.hyo = *B.hyodysenteriae*; B.pilo = *B.pilosicoli*

Group 1				
A				
Species distribution				
		Culture DNA % (n)		
*B.hyodysenteriae*		19.5% (49)		
*B.pilosicoli*		10% (25)		
apathogen		36.6% (92)		
mixes		33.9% (85)		
*B.hyo* + apathogen		6.8% (17)		
*B.pilo* + apathogen		23.1% (58)		
*B.hyo* + *B.pilo*		2.4% (6)		
*B.hyo* + *B.pilo* + apathogen		1.6% (4)		
Total samples		total =251		
B				
Multiplex vs. Duplex on fecal culture (*n* = 251)				
		Duplex PCR		Kappa index
		+	-	
Multiplex qPCR				
*B.hyodysenteriae*	+	71	5	0.943
	-	1	174	
*B.pilosicoli*	+	92	1	0.933
	-	7	151	

In 91 samples (36.3%) the multiplex qPCR was able to demonstrate the presence of *Brachyspira* i.e. the triplet of apathogenic *Brachyspira spp.* Another panel of 86 samples from the multiplex qPCR analysis exhibited different combinations of mixed infections with *B.hyodysenteriae*, *B.pilosicoli* and apathogenic *Brachyspira.*spp. With the multiplex assay ten mixed infections of *B.hyodysenteriae* and *B.pilosicoli* (4.0%) were detected. The duplex PCR was able to confirm four cases (1.6%) of these *B.hyodysenteriae* and *B.pilosicoli* mix infections (Table 3).

#### Comparative PCR analysis using DNA from cultured samples and from swabs

**Fig. 2:**
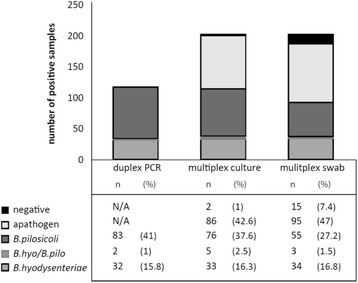
Comparison of result between the duplex PCR and the qPCR with culture derived DNA and swab DNA (Group 2). N/A: not applicable

A total of 202 fecal swabs were examined for *Brachyspira* spp. by comparative PCR analysis of DNA from culture harvests and DNA directly extracted from the swabs (Fig. 1, Group 2). Using DNA from culture harvests, both PCR methods were again in excellent agreement in detecting *B. hyodysenteriae* and *B. pilosicoli* (κ-values of 0.932 and 0.928, Table 4). A direct comparison of both PCR methods on direct swab DNA was not feasible since the vast majority of samples analyzed by duplex PCR were negative due to the substantially lower LOD in comparison to the multiplex qPCR. Comparative analysis of both swab and culture harvest DNA in the multiplex qPCR revealed that the detection rate of *B. hyodysenteriae* was 18% (37–38 positive) in either type of specimen. *B. pilosicoli* was detected in 81 DNA samples from culture harvests (40.1%), whereas swab DNA was found positive in 58 cases (28.7%). The results obtained by comparative multiplex qPCR analysis of swab and culture harvest DNA were in large agreement regarding the detection rate of *B. hyodysenteriae* and the triplet of apathogenic *Brachyspira* spp. alone or in different combinations (Fig. 2). Overall, the detection rates for *B. hyodysenteriae* and *B. pilosicoli* were ranked as “almost perfect” and in “substantial” agreement, respectively (κ-values of 0.853 and 0.643, Table 4). In addition, DNA from 75 fecal swabs, which were evaluated *Brachyspira* negative by microscopy evaluation of culture growth, were analyzed with the multiplex qPCR. Fifty-one of these samples were negative in the multiplex qPCR analysis, however, 23 samples (30.7%) were positive for apathogen *Brachyspira* spp. and one sample was positive for *B. pilosicoli* and apathogen spp. with a C_t_-value close to the threshold (C_t_: 37.8 for *B. pilosicoli)*. Two additional samples from the same herd as the *B. pilosicoli* positive sample were submitted to our laboratory for analysis. Both samples were evaluated *B.pilosicoli* positive by duplex PCR and multiplex qPCR.

**Table 4:** Species distribution of samples in Group 2 (A). Agreement between the multiplex qPCR and the duplex PCR using the kappa index (Group 2) (B). The second panel displays the detection of *B.hyodysenteriae* and *B.pilosicoli* and the agreement between culture derived DNA vs swab derived DNA using the multiplex qPCR assay (C)

Group 2				
A				
Species distribution				
		Culture DNA % (n)		
*B.hyodysenteriae*		10.4% (21)		
*B.pilosicoli*		8.9% (18)		
apathogen		47% (95)		
negative for *Brachyspira*		7.4% (15)		
mixes		26.3% (53)		
*B.hyo* + apathogen		6.5% (13)		
*B.pilo* + apathogen		18% (37)		
*B.hyo* + *B.pilo*		0.5% (1)		
*B.hyo* + *B.pilo* + apathogen		1% (2)		
Total samples		total =202		
B				
Multiplex vs. Duplex on fecal culture (*n* = 202)				
		Duplex PCR		Kappa index
		+	-	
Multiplex qPCR culture				
*B.hyodysenteriae*	+	34	4	0.932
	-	0	164	
*B. pilosicoli*	+	79	2	0.928
	-	5	116	
C				
Multiplex qPCR culture vs. swabs (*n* = 202)				
		Culture		Kappa index
		+	-	
Swabs				
*B.hyodysenteriae*	+	33	4	0.853
	-	5	160	
*B.pilosicoli*	+	53	5	0.643
	-	28	116

## Discussion

In this study, a total of 453 fecal samples from pigs originated from a cross-section of the Swiss cantons in which pig breeding and pig farming is carried out (Fig. 1), were analyzed for *Brachyspira* spp. to evaluate a novel multiplex qPCR, designed to simultaneously detect *B. hyodysenteriae*, *B. pilosicoli* and a triplet of apathogenic *Brachyspira* spp. *(B. intermedia, B. murdochii, B. innocens*). Using spirochetal DNA from fecal anaerobic cultures as a target, the multiplex qPCR displayed an almost perfect agreement with the reference duplex PCR with kappa index values of 0.943 and 0.933 for *B.hyodysenteriae* and *B.pilosicoli*, respectively. Regarding the advantage of identifying the two significant pathogenic *Brachyspira* spp. from cultures of porcine fecal specimens in one PCR run, the novel multiplex qPCR can definitively replace the more labor-intensive duplex PCR. The latter one is relying on the amplification of two different genes (*nox* and 16S rDNA gene) for the detection of *B.hyodysenteriae* and *B.pilosicoli* [14]. Our novel assay on the contrary is based on one single target sequence (namely the 23S rDNA gene), a fact which increases the efficiency of the PCR and additionally avoids competition for the PCR reagents between different targets. Traditionally 16S rDNA sequencing has been used for the differentiation between different bacteria species, but members of the genus *Brachyspira* have been reported difficult to differentiate solely based on this gene sequence [23]. The 23S rDNA on other hand, comprises of longer regions of hypervariability [24] and has previously been used for specific detection of *B. hyodysenteriae* and two other groups of weakly beta-hemolytic procine *Brachyspira* [25]. Another benefit of the multiplex qPCR is the internal control which informs about PCR inhibitor factors, and thereby helps avoiding false-negative samples.

In other studies on the detection of porcine *Brachyspira* using a variety of PCR protocols minimal detection limits between 10^2^ and 10^4^ cells or DNA copies per gram specimen (depending on study) has been demonstrated [25–27]. Even with a moderate analytic sensitivity a PCR should reliably identify pigs with clinical SD. However, the detection of the epidemiologically important asymptomatic carriers, which according to experience shed only low numbers of *Brachyspira,* is severely limited [13]. The analytic sensitivity of our multiplex qPCR was determined to 5–10 GE which corresponds to a detectable minimum bacterial load of minimum 6 × 10^2^ and on average 1 × 10^3^ GE per g specimen. The black hole quencher used in this assay is providing a low signal-to-noise ratio and thereby also providing a higher sensitivity compared to traditional probes with a secondary fluorescent dye as quencher. Thus, in comparison with suitable data from the literature, our multiplex PCR shows a promising analytic sensitivity which should be adequate to identify *Brachyspira* spp. in DNA directly extracted from fecal swabs. Interestingly, *Brachyspira* spp. patterns obtained by PCR analysis of culture-derived DNA only slightly differed from the patterns obtained from the corresponding swab DNA with exception of *B. pilosicoli* (Fig. 2). DNA samples derived directly from fecal swabs are expected to display the natural distribution of *Brachyspira* spp. By comparison, DNA from culture harvests may not reflect the original brachyspiral spectrum in a fecal specimen since the culture conditions may either stimulate or even delay the growth rate of a single *Brachyspira* sp. in a brachyspiral mixture. Accordingly, *B.pilosicoli* appeared to be more susceptible to a cultural enrichment. In our study *B.pilosicoli* either alone or in mixed infection was detected at a significant higher frequency in DNA samples from culture harvests than in swab DNA (81 vs. 58, *p* < 0.05, Chi square test). It is however important to consider that the swabs used in this study had already been used streaking out on TSA, consequently less material is available for the direct swab DNA extraction. Extracting DNA from the swabs directly after delivery might improve the result and lead to an increased concordance with the cultured DNA analyzed with the reference duplex PCR or our novel multiplex qPCR. As a step in the evaluation of the multiplex qPCR we tested 75 swabs evaluated by culture to be *Brachyspira* negative. Thirty percent of the samples were positive for *Brachyspira* spp. when analyzed with our assay. This high number of *Brachyspira* spp. findings in culture negative swabs confirms the high sensitivity of the novel multiplex qPCR even in comparison with culture.

With PCR analysis of DNA from swabs, without culturing the samples, the result can be delivered already after 1 day. This is a substantial improvement compared to the conventional microbiological culture methods where 3 to 6 days of culture is needed until DNA can be extracted and the analysis for pathogenic *Brachyspira* spp. can be achieved. Both the culture and swab samples methods show high specificity, however, the culture coupled qPCR has the highest sensitivity, especially in the case of *B.pilosicoli* detection due to its fast growth rate in culture. Therefore, we recommend these two analysis approaches in different setups: high-throughput screening of fecal swabs and the culture-coupled approach to identify *B.hyodysenteriae* infected individuals within qPCR positive herds with the purpose to establish pathogen *Brachyspira* strains e.g. for antimicrobial susceptibility testing. We are currently evaluating the effect of a short incubated enrichment broth on further increasing the sensitivity of the swab-based multiplex qPCR for *B.hyodysenteriae* and *B.pilosicoli* to close the small, but still evident, sensitivity gap between culture-harvest and swab DNA analysis.

## Conclusion

In comparison to a widely used reference PCR a novel multiplex qPCR targeting a 128 bp region on the 23S rDNA gene allows the sensitive simultaneous detection of *B. hyodysenteriae* and *B. pilosicoli* and three apathogenic *Brachyspira* spp in fecal swabs. The multiplex qPCR provides more detailed insights into the composition of the porcine brachyspiral fecal due to the concurrent detection of pathogenic and apathogenic *Brachyspira* spp. We propose this assay as a robust diagnostic tool with the ability to decrease the work load and improve the diagnostic results readout.

## Additional files

Additional file 1:
**Figure S1.** Consensus sequence alignment of the target DNA region within 23S ribosomal DNA. Primers (Brachy primer for. and Brachy primer rev.) on the target DNA are marked in grey. The probe for *B. hyodysenteriae* (Probe_hyo) is highlighted in yellow, the probe for *B. pilosicoli* (Probe_pilo) in purple, and the probe for the *B. intermedia/B. innocens/B. murdochii* (probe inter) in green. Differences in single residues are marked in red. (PDF 112 kb)
Additional file 2:
**Figure S2.** A panel of 26 spirochetal and non-spirochetal bacteria used for specificity testing of the multiplex qPCR. ATCC, American Type Culture Collection; DSM, German Collection of Microorganisms [Deutsche Sammlung von Mikrooganismen]. (PDF 258 kb)
Additional file 3:
**Figure S3.** Plotting of standard curves for the three probes. (PDF 67 kb)

## Abbreviations

PIS: Porcine intestinal spirochaetosis
SD: Swine dysentery

## Footnotes

1 Footnote: Strains were kindly provided by Dr. Judith Rhode, Institute for Microbiology, University of Veterinary Medicine Hanover, Hanover, Germany.
