# Supplementary material for: A novel multiplex qPCR targeting 23S rDNA for diagnosis of swine dysentery and porcine intestinal spirochaetosis
Source: BMC Vet Res. 2017 Feb 7;13:42. doi: 10.1186/s12917-016-0939-6 (PMC5297149; doi:10.1186/s12917-016-0939-6)
Supplement: Additional file 1: — Figure S1. Consensus sequence alignment of the target DNA region within 23S ribosomal DNA. Primers (Brachy primer for. and Brachy primer rev.) on the target DNA are marked in grey. The probe for B. hyodysenteriae (Probe_hyo) is highlighted in yellow, the probe for B. pilosicoli (Probe_pilo) in purple, and the probe for the B. intermedia/B. innocens/B. murdochii (probe inter) in green. Differences in single residues are marked in red. (PDF 112 kb) [file 12917_2016_939_MOESM1_ESM.pdf]

```
                           20                    40                    60                    80
B.hyodesenteriae  TCTGCCTAGG GTTAGTCGAC CCCTAAGATG AGGCTGAAAA GCGTAGTCGA TGGGAAACAG GTAAATATTC CTGTACTATG 80
B.pilosicoli      TCTGCCTAGG GTTAGTCGAC CCCTAAGATG AGGCTGAAAA GCGTAGTCGA TGGGAAACAG GTAAATATTC CTGTACTATA 80
B.intermedia      TCTGCCTAGG GTTAGTCGAC CCCTAAGATG AGGCTGAAAA GCGTAGTCGA TGGGAAACAG GTAAATATTC CTGTACTATG 80
B. innocens       TCTGCCTAGG GTTAGTCGAC CCCTAAGATG AGGCTGAAAA GCGTAGTCGA TGGGAAACAG GTAAATATTC CTGTACTATG 80
B.murdochii       TCTGCCTAGG GTTAGTCGAC CCCTAAGATG AGGCTGAAAA GCGTAGTCGA TGGGAAACAG GTAAATATTC CTGTACTATG 80

                          100                   120                   140                   160
B.hyodesenteriae  ATATGTTTCG ATGGAATGAC ACAGATTGTT TATGTACGCG TTAGATTGGT AGATAACGTC AAACAGTTTA GACTTGAGGT 160
B.pilosicoli      GTATGTTTCG ATGGAATGAC ACAGATTGTT TGCGTACGCG AGGTGATGGT TATCCTCGTC GAATAGCCGA GACTTGTGGT 160
B.intermedia      ATATGTTTCG ATGGAATGAC ACAGATTGTT TGCGTACGCG TTAGATTGGT AGATAACGTC AAACAGTTTA GACTTGTGAT 160
B. innocens       ATATGTTTCG ATGGAATGAC ACAGATTGTT TACGTACGCG TTAGATTGGT AGATAACGTC AAACAGTTTA GACTTGTGAT 160
B.murdochii       ATATGTTTCG ATGGAATGAC ACAGATTGTT TACGTACGCG TTAGATTGGT AGATAACGTC AAACAGTTTA GACTTGTGAT 160

                          180                   200                   220                   240
B.hyodesenteriae  GAGTCAAATG CTTGCTTCTT TAAGGTTAAG GCTGGATAGT GACTGGGCTT TCGGGTTCAG GAAGTTGCAT GAGCTAGGCT 240
B.pilosicoli      GAGTAAAATG CTTGCTGCTT TAAGGTTGAG G-TGGATAGT GACTGGGCTT TCGGGTTCAG GAAGTTGCGT GAATTAGGCT 239
B.intermedia      GAGTAAAATG CTTGTTGCTT TAAGGTTGAG GCTGGATAGT GACTGGGCTT TCGGGTTCAG GAAGTTGCGT GAGCTAGGCT 240
B. innocens       GAGTAAAATG CTTGTTGCTT TAAGGTTGAG GCTGGATAGT GACTGGGCTT TCGGGTTCAG GAAGTTGCGT GAGCTAGGCT 240
B.murdochii       GAGTAAAATG CTTGTTGCTT TAAGGTTGAG GCTGGATAGT GACTGGGCTT TCGGGTTCAG GAAGTTGCGT GAGCTAGGCT 240

                          260                   280                   300
B.hyodesenteriae  GTCGAGAAAT AATTTCTAAG GTTAGGCATG TCATAATCGT ACCGCAAACC GACACAGGTG GGTGA 305
B.pilosicoli      GTTTAGAAAT AATTCCTAAG GTTAGGCATA TTATAATTGT ACCGCAAACC GACACAGGTG GGTGA 304
B.intermedia      GTCGAGAAAT AATTTCTAAG GTTAGGCATG TCGTAATCGT ACCGCAAACC GACACAGGTG GGTGA 305
B. innocens       GTCGAGAAAT AATTTTTAAG GTTAGGCATG TCATAATTGT ACCGCAAACC GACACAGGTG GGTGA 305
B.murdochii       GTCGAGAAAT AATTTCTAAG GTTAGGCATG TCATAATCGT ACCGCAAACC GACACAGTTG GGTGA 305
```
