# Supplementary material for: A novel multiplex qPCR targeting 23S rDNA for diagnosis of swine dysentery and porcine intestinal spirochaetosis
Source: BMC Vet Res. 2017 Feb 7;13:42. doi: 10.1186/s12917-016-0939-6 (PMC5297149; doi:10.1186/s12917-016-0939-6)
Supplement: Additional file 2: — Figure S2. A panel of 26 spirochetal and non-spirochetal bacteria used for specificity testing of the multiplex qPCR. ATCC, American Type Culture Collection; DSM, German Collection of Microorganisms [Deutsche Sammlung von Mikrooganismen]. (PDF 258 kb) [file 12917_2016_939_MOESM2_ESM.pdf]

**Organism**	**Source/Strain**
Borrelia burgdorferi B31	ATCC 35210
Borrelia heimsii	ATCC 35209
Klebsiella pneumoniae	clinical isolate
Trueperella pyogenes	DSM 20630
Yersinia enterocolitica	DSM 11502
Streptococcus equi ssp. equi	clinical isolate
Pasteurella multocida	clinical isolate
Aeromonas hydrophila	DSM 6173
Campylobacter coli	DSM 4689
Campylobacter jejuni	DSM 4688
Clostridium perfringens Typ A	DSM 756
Pseudomonas aeruginosa	ATCC 27853
Staphylococcus aureus	ATCC 25923
Enterococcus faecalis	ATCC 29212
Escherichia coli	clinical isolate
Staphylococcus aureus, MRSA	ATCC 43300
Enteroccoccus faecalis, VRE	ATCC 51299
Corynebacterium pseudotuberculosis	ATCC 19410
Staphylococcus intermedius	DSM 20373
Staphylococcus pseudintermedius	DSM 21284
Actinobacillus pleuropneumoniae	DSM 13472
Haemophilus parasuis	DSM 21448
Rhodococcus equi	DSM 20307
Streptococcus agalactiae	DSM 2134
Corynebacterium renale	DSM 20688
Bordetella bronchiseptica	clinical isolate
