# Supplementary figures and images for: A novel multiplex qPCR targeting 23S rDNA for diagnosis of swine dysentery and porcine intestinal spirochaetosis
Source: BMC Vet Res. 2017 Feb 7;13:42. doi: 10.1186/s12917-016-0939-6 (PMC5297149; doi:10.1186/s12917-016-0939-6)

Appendix Fig. A.3 One and a half column, 140mm

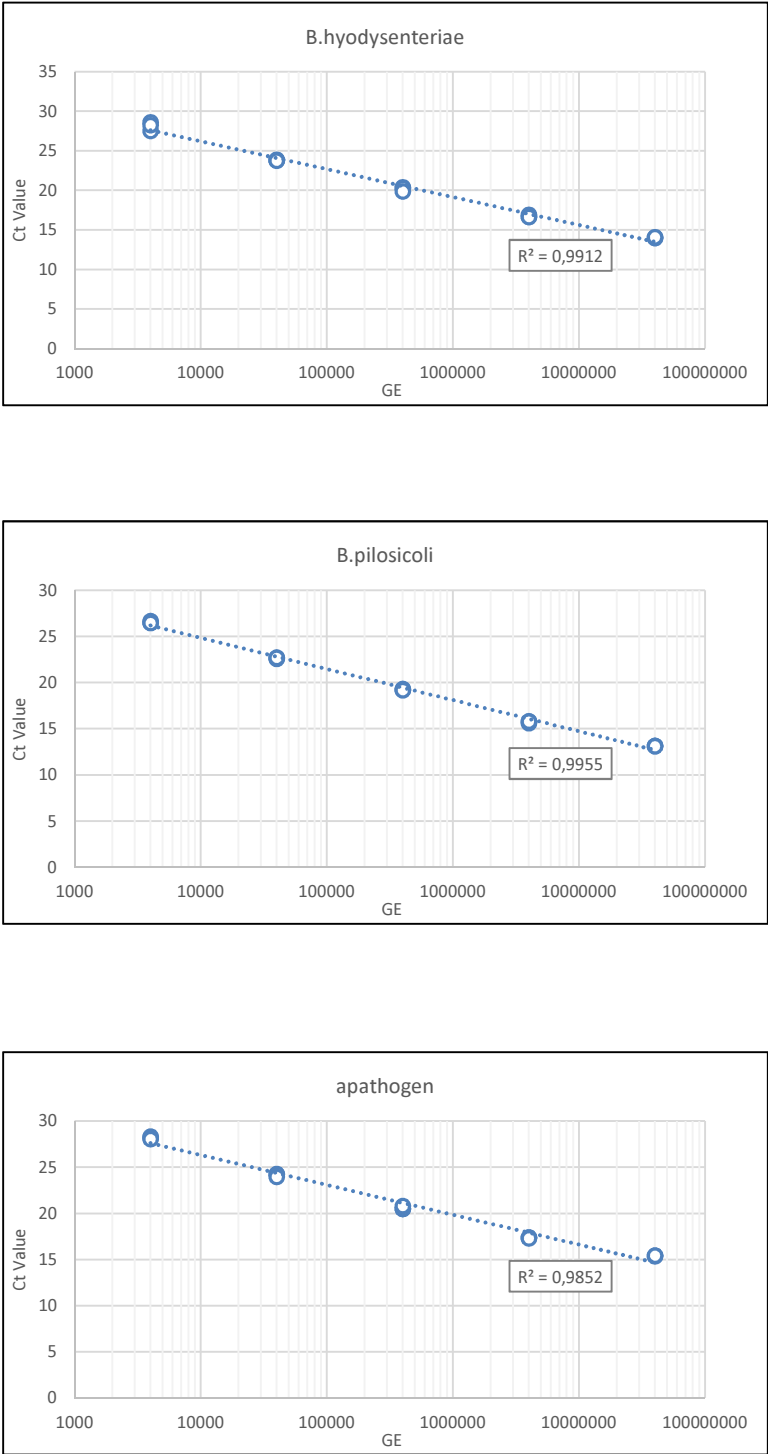

Fig. A.3 Plotting of standard curves for the three probes.

Supplement: Additional file 3: — Figure S3. Plotting of standard curves for the three probes. (PDF 67 kb) [file 12917_2016_939_MOESM3_ESM.pdf]
